# NPT100-18A rescues mitochondrial oxidative stress and neuronal degeneration in human iPSC-based Parkinson’s model

**DOI:** 10.1186/s12868-025-00926-y

**Published:** 2025-01-28

**Authors:** Julian E. Alecu, Veronika Sigutova, Razvan-Marius Brazdis, Sandra Lörentz, Marios Evangelos Bogiongko, Anara Nursaitova, Martin Regensburger, Laurent Roybon, Kerstin M. Galler, Wolfgang Wrasidlo, Beate Winner, Iryna Prots

**Affiliations:** 1 Department of Stem Cell Biology, University Hospital Erlangen, Friedrich-Alexander University of Erlangen-Nürnberg, Erlangen, Germany; 2 Department of Psychiatry and Psychotherapy, University Hospital Erlangen, Friedrich-Alexander University of Erlangen-Nürnberg, Erlangen, Germany; 3 Department of Molecular Neurology, University Hospital Erlangen, Friedrich-Alexander University of Erlangen- Nürnberg, Erlangen, Germany; 4 Department of Neurodegenerative Science, the MiND program, Van Andel Institute, Grand Rapids, MI USA; 5 Department of Operative Dentistry and Periodontology, University Hospital Erlangen, Friedrich-Alexander University of Erlangen-Nürnberg, Erlangen, Germany; 6 Neuropore Therapies, Inc, San Diego, CA USA; 7 Department of Neuroscience, University of California, San Diego, La Jolla, CA USA; 8 Center for Rare Diseases Erlangen (ZSEER), University Hospital Erlangen, Friedrich-Alexander University of Erlangen-Nürnberg, Erlangen, Germany

**Keywords:** Parkinson’s disease, Alpha-synuclein, Aggregation, Oxidative stress, ROS, iPSC, Dopaminergic neurons, Mitochondria, NPT100-18A

## Abstract

**Background:**

Parkinson’s disease (PD) is a neurodegenerative disorder characterized by protein aggregates mostly consisting of misfolded alpha-synuclein (αSyn). Progressive degeneration of midbrain dopaminergic neurons (mDANs) and nigrostriatal projections results in severe motor symptoms. While the preferential loss of mDANs has not been fully understood yet, the cell type-specific vulnerability has been linked to a unique intracellular milieu, influenced by dopamine metabolism, high demand for mitochondrial activity, and increased level of oxidative stress (OS). These factors have been shown to adversely impact αSyn aggregation. Reciprocally, αSyn aggregates, in particular oligomers, can impair mitochondrial functions and exacerbate OS. Recent drug-discovery studies have identified a series of small molecules, including NPT100-18A, which reduce αSyn oligomerization by preventing misfolding and dimerization. NPT100-18A and structurally similar compounds (such as NPT200-11/UCB0599, currently being assessed in clinical studies) point towards a promising new approach for disease-modification.

**Methods:**

Induced pluripotent stem cell (iPSC)-derived mDANs from PD patients with a monoallelic *SNCA* locus duplication and unaffected controls were treated with NPT100-18A. αSyn aggregation was evaluated biochemically and reactive oxygen species (ROS) levels were assessed in living mDANs using fluorescent dyes. Adenosine triphosphate (ATP) levels were measured using a luminescence-based assay, and neuronal cell death was evaluated by immunocytochemistry.

**Results:**

Compared to controls, patient-derived mDANs exhibited higher cytoplasmic and mitochondrial ROS probe levels, reduced ATP-related signals, and increased activation of caspase-3, reflecting early neuronal cell death. NPT100-18A-treatment rescued cleaved caspase-3 levels to control levels and, importantly, attenuated mitochondrial oxidative stress probe levels in a compartment-specific manner and, at higher concentrations, increased ATP signals.

**Conclusions:**

Our findings demonstrate that NPT100-18A limits neuronal degeneration in a human in vitro model of PD. In addition, we provide first mechanistic insights into how a compartment-specific antioxidant effect in mitochondria might contribute to the neuroprotective effects of NPT100-18A.

**Supplementary Information:**

The online version contains supplementary material available at 10.1186/s12868-025-00926-y.

## Background

Parkinson’s disease (PD) is a complex and progressive neurodegenerative disease with a markedly increasing incidence and global disease burden [1, 2]. PD is characterized by early and prominent loss of midbrain dopaminergic neurons (mDANs), resulting in disruption of nigrostriatal circuits. This results in motor symptoms encompassing hypo- and bradykinesia, muscular rigidity, and resting tremor [3]. Degeneration of mDANs is tightly connected to the intracellular accumulation of alpha-synuclein (αSyn)-containing protein aggregates, which reflect a neuropathological hallmark of PD [4].

Under physiological conditions, αSyn, encoded by *SNCA*, acts as a regulator of synaptic vesicle release and is enriched in presynaptic terminals. Within this compartment, αSyn cycles between a monomeric, highly soluble, and a membrane-bound multimeric state [5–9]. Increased expression of wild-type (WT) αSyn caused by copy number multiplications of the *SNCA* locus and single-nucleotide polymorphisms in non-coding enhancers of *SNCA*, have been linked to familial and idiopathic forms of PD [10–12]. Under these conditions, αSyn is prone to aggregate via oligomeric intermediate states towards large fibrillar aggregates, which solidify as Lewy bodies and Lewy neurites [13–16].

αSyn oligomers, a form of particularly toxic aggregates, are capable of impairing a multitude of cellular pathways including autophagy, proteasomal clearance, vesicle transport, as well as the endoplasmic reticulum (ER) and mitochondria [9, 17–23]. In particular, the latter appear to be a preferential target of αSyn-mediated toxic effects [20, 24–27]. Dysfunctional mitochondria in turn act as catalysts for αSyn aggregation and neuronal cell death through reduced ATP regeneration, release of calcium, and excessive generation of reactive oxygen species (ROS) [20, 28]. This crucial link between αSyn aggregation and mitochondrial dysfunction is further reflected by familial forms of PD, caused by pathologic variants in genes (e.g. *DJ-1*, *PINK1,* and *PRKN*) that regulate the mitochondrial oxidative stress (OS) response [29, 30]. In addition, we have previously shown that increased αSyn aggregation and ROS levels determine the cell type-specific vulnerability of mDANs in PD [31].

At present, no disease-modifying therapy for PD is available. However, important preclinical progress in targeting disease-relevant pathways has been made in recent years. Among the most promising approaches is NPT100-18A, a *de novo* compound identified in a structure-based drug-discovery [32]. The peptidomimetic small molecule inhibits protein-protein interaction between αSyn residues 96–102 and residues 80–90 of a complementary αSyn monomer. This prevents dimerization and propagation to toxic oligomers [32]. NPT100-18A and structurally similar compounds such as NPT200-11/UCB0599 (currently assessed in clinical trials: NCT02606682 [study start 2015-07], NCT04658186 [study start 2020-12], NCT04875962 [study start 2019-05], NCT05543252 [study start 2022-08]; https://www.clinicaltrials.gov/) have been previously shown to reduce αSyn pathology, astrogliosis, and improve behavioral deficits in rodent in vivo models of PD. Furthermore, NPT100-18A treatment restored intact neurite morphology and mitochondrial axonal transport in human stem cell-based in vitro neuronal models expressing mutants of αSyn [23, 32–34]. However, to date, the molecular actions of NPT100-18A in human WT αSyn-expressing dopaminergic neurons remain unclear.

This study investigates the effects of NPT100-18A in a human in vitro model of PD using induced pluripotent stem cell (iPSC)-derived neurons from patients with a monoallelic *SNCA* locus duplication. To study the effects of the compound on the aforementioned molecular drivers of PD neuropathology, αSyn, and mitochondrial dysfunction, as well as overall neuronal survival, we evaluated cellular compartment-specific oxidative stress, ATP levels, and neuronal viability in mDANs differentiated from PD patient- and control-derived iPSCs.

## Methods

### Cells and cell culture

A total of five human iPSC lines were used. Two iPSC lines from PD patients bearing heterozygous *SNCA* locus duplication were kindly provided by Prof. Galasko (line SDi1-R-C: clones SDi1-R-C3 and SDi1-R-C11 [23, 31]) and Prof. Roybon (line CSC-1: clones CSC-1A and CSC-1D [31, 35]). IPSCs from three age-matched healthy Caucasian individuals with no history of neurologic disease served as controls (cell lines UKERi82A-S1_017, UKERi33Q-R1-06, and UKERiO3H-R1: clones UKERiO3H-R1-001 and UKERiO3H-R1-005) and have been previously characterized [23, 31, 36]. IPSCs were differentiated through neural precursor cells (NPCs) into mDANs using a fibroblast growth factor 8 (FGF-8)- and small molecule-based midbrain protocol as previously described [31, 37]. NPCs were seeded onto Geltrex-coated 12-well plates and differentiated into mDANs using differentiation medium (50% DMEM/F12, 50% Neurobasal Medium, N2 [0.5x], B27 [0.5x]), supplemented with FGF-8b (100 ng/ml), purmorphamine (PMA, 1.2 µM), and ascorbic acid (200 nM). For maturation, cells were dissociated using accutase, seeded onto polyornithine/laminin-coated plates at 75 × 10^3^/cm^2^ cell density and cultured for 14 days in differentiation medium supplemented with transforming growth factor beta-3 (TGF-β3, 1 ng/ml), glial cell-line derived neurotrophic factor (GDNF, 10 ng/ml), brain-derived neurotrophic factor (BDNF, 10 ng/ml), ascorbic acid (200 nM), and dibutyryl-cAMP (500 µM). During the first two days of maturation, 0.6 µM PMA was added to the medium. Contamination of the cell lines with mycoplasma was excluded routinely, using a PCR-based mycoplasma test. Reagents used are listed in Table S1.

### NPT100-18A compound and treatment regimen

NPT100-18A is a *de novo* peptidomimetic compound with a cyclic pyrimido-pyrazine scaffold developed and previously described by Wrasidlo et al. [32]. NPT100-18A has previously been extensively characterized and shown to reduce human αSyn aggregation in iPSC-derived neurons and other cellular PD modes in vitro and in vivo [23, 32–34]. NPT100-18A was kindly provided by W. Wrasidlo and chemical purity was verified via LC-MS. The compound was dissolved in DMSO at the concentration of 20 µM. NPT100-18A was added to the medium at a final concentration of 10 nM (or 100 nM and 1 µM for ATP dose-response measurements) starting from the first day of differentiation until the end of the maturation period. In parallel, mDANs were cultured under vehicle condition: differentiation/maturation medium with DMSO.

### αSyn solubility assay and immunoblotting

Cells were manually homogenized in 1% Triton-X100-containing buffer (50 mM Tris-HCl pH 8.0, 150 mM NaCl, 1 mM EDTA, 1.5 mM MgCl2 with protease and phosphatase inhibitors). The lysate was separated into soluble and insoluble fractions by 20 min (min) centrifugation (18000 x g, 4 °C) and the insoluble pellet was resuspended in 0.5 M urea/5% SDS. For the soluble fraction, 10 µg of total protein in a total volume of 5 µl was spotted onto 0.2 μm nitrocellulose membrane, while for the insoluble fraction, 5 µl of the lysate was spotted. Membranes were air-dried for 2 hours (h) and subsequently fixed with 4% paraformaldehyde (PFA) for 20 min at room temperature (RT) to improve detection [38]. Membranes were blocked in 5% non-fat dry milk in Tris-buffered saline (TBS) containing 0.1% Tween 20 (TBST) for 1 h at RT and incubated with primary antibodies against aggregated and total αSyn (Table S1) diluted in 3% bovine serum albumin in TBST, followed by the appropriate horseradish peroxidase (HRP)-conjugated secondary antibodies. Chemiluminescent signal was detected with ECL Select on the Gel Doc XR system. Total protein loading was controlled by staining the membranes using 0.008% Direct Blue 71, 40% ethanol, and 10% acetic acid for 5 min at RT, followed by de-staining with 150 mM sodium bicarbonate in 47.5% ethanol. Images were quantified using the Fiji software. The conformer and total αSyn signals were normalized to total protein and the conformer αSyn signal was subsequently normalized to total αSyn, followed by calculating the fraction of αSyn conformers in soluble and insoluble fractions. Antibodies and reagents used are listed in Table S1.

### Immunocytochemistry and image acquisition

Immunocytochemistry and image acquisition were done as previously described [31, 36]. Briefly, cells were fixed using 4% PFA for 30 min at 37°C, permeabilized with ice-cold ethanol and acetic acid (2:1), washed three times with Dulbecco’s phosphate-buffered saline (DPBS), and subsequently permeabilized and blocked using blocking solution (0.3% Triton-X100, 5% donkey serum in DPBS). Primary antibodies (diluted in blocking solution) were added overnight at 4°C. Fluorochrome-conjugated secondary antibodies were added for 1 h at RT. Cell nuclei were stained with 1 µg/ml 4′,6-diamidino-2-phenylindole (DAPI). Coverslips with stained cells were mounted on glass microscope slides using Aqua Polymount. Images were acquired on an Axio Observer Z1 inverted fluorescence microscope using Zen Black software. A Z-stack of ten optical sections was captured for each field, and a maximum intensity projection was generated for subsequent analysis. Images were evaluated blinded with regard to genotype and treatment of the cell line using the cell counter and blind analysis tool plugin for Fiji software [39]. Three independent differentiation rounds were done for each NPC line and at least three images were evaluated for each differentiation to assess neuronal cell death. Antibodies and reagents used are listed in Table S1.

### ROS measurements

Cytosolic and mitochondrial ROS levels were quantified in living mDANs as previously described [31]. Briefly, NPCs were seeded at a density of 50 × 10^3^ cells per well in black 96-well plates with transparent flat bottom coated with polyornithine/laminin. On the final maturation day, mDANs were stained with either CellROX Green- or MitoSOX Red fluorescent dyes for detection of total intracellular ROS or mitochondrial superoxide, respectively. CellROX was added directly to the culture media at a concentration of 5 µM for 30 min at 37°C. Prior to incubation with MitoSOX, neurons were washed once with warm DPBS (with Ca^2+^/Mg^2+^). MitoSOX (diluted in DPBS with Ca^2+^/Mg^2+^) was then added to the cells to a final concentration of 5 µM for 10 min at 37°C. After staining with CellRox or MitoSOX, DAPI (10 µg/ml, diluted in DPBS) was added for 5 min at 37°C and then cells were washed twice with warm DPBS. CellROX, MitoSOX, and DAPI fluorescence intensities (FIs) were measured on a CLARIOstar Plus microplate reader (BMG Labtech) using the following excitation/emission wavelengths: CellROX – 500 − 20/530 − 25 nm; MitoSOX – 510 − 15/580 − 20 nm; DAPI – 360 − 20/460 − 30 nm. Separate staining with MitoSOX and CellRox as well as separate acquisition of fluorescence channels were used to prevent spectral crosstalk in this set of experiments. A matrix scan protocol was used, measuring FIs at 80 sites per well. CellROX and MitoSOX FIs were normalized to DAPI for each site followed by normalization to the mean values of control line C1 for comparison of control and patient neurons, or normalization to the mean value of the respective vehicle control condition for NPT100-18A treatment experiments. NPC lines were differentiated in four independent experiments. For each differentiation, neurons were cultured in quadruplicates at least. Reagents used are listed in Table S1. In order to test the sensitivity of the MitoSOX probe to changes in ROS concentrations, an H_2_O_2_ challenge with 300 µM H_2_O_2_ for 2 h in mature P1.1 neurons was performed.

### ATP measurements

Relative ATP levels in living mDANs were assessed as previously described [23]. NPCs were seeded at a density of 50 × 10^3^ cells per well in white opaque 96-well plates coated with polyornithine/laminin. ATP measurements were performed using the luminescence-based CellTiter-Glo 2.0 Cell Viability Assay kit according to the manufacturer’s instructions. Luminescence was measured using a LUMIstar Omega microplate reader. In parallel, neurons were differentiated under the same conditions in black 96-well plates with transparent flat bottom for viability testing using Image IT DEAD Green Viability Stain. Neurons were incubated with 100 nM IT DEAD Green reagent for 30 min at 37°C, fixed using 4% PFA, and stained with DAPI (10 µg/ml diluted in DPBS). NPC lines were differentiated in three independent rounds. For each differentiation round, cells were cultured in duplicates at least. Three images were acquired for each well using an Axio Observer Z1 inverted fluorescence microscope. Relative ATP amount per cell was estimated by normalization of ATP-related luciferase signal to the percentage of viable cells followed by normalization to the mean values of control line C1 for comparison of control and patient neurons, or normalization to the mean value of the respective vehicle control condition for NPT100-18A treatment experiments. Reagents used are listed in Table S1.

### Statistics

Normality of distributions was assessed using D’Agostino-Pearson test and graphical methods and correspondingly, means and standard deviations are reported for continuous variables. Differences between groups and conditions were evaluated using two-tailed nested t-test with technical replicates (n) nested within separate differentiations (N) or, where indicated, technical replicates nested within differentiations and cells lines. Total intracellular ROS probe intensities in DMSO/NPT100-18A-treated mDANs and the percentage of DMSO/NPT100-18A-treated cleaved caspase-3 (cCasp3)/tyrosine hydroxylase (TH)-double positive neurons were log-transformed to meet model assumptions. Statistical analyses were performed using Prism version 10.3.1 (GraphPad Prism). All statistical tests were two-sided and *P* ≤ 0.05 was considered significant. *P* values are denoted as follows: *P* ≤ 0.05 (*), *P* ≤ 0.01 (**), *P* ≤ 0.001 (***) and *P* ≤ 0.0001 (****).

## Results

### Increased αSyn aggregation coincides with increased ROS probe intensities and mitochondrial dysfunction in PD patient-derived mDANs

**Fig. 1:**
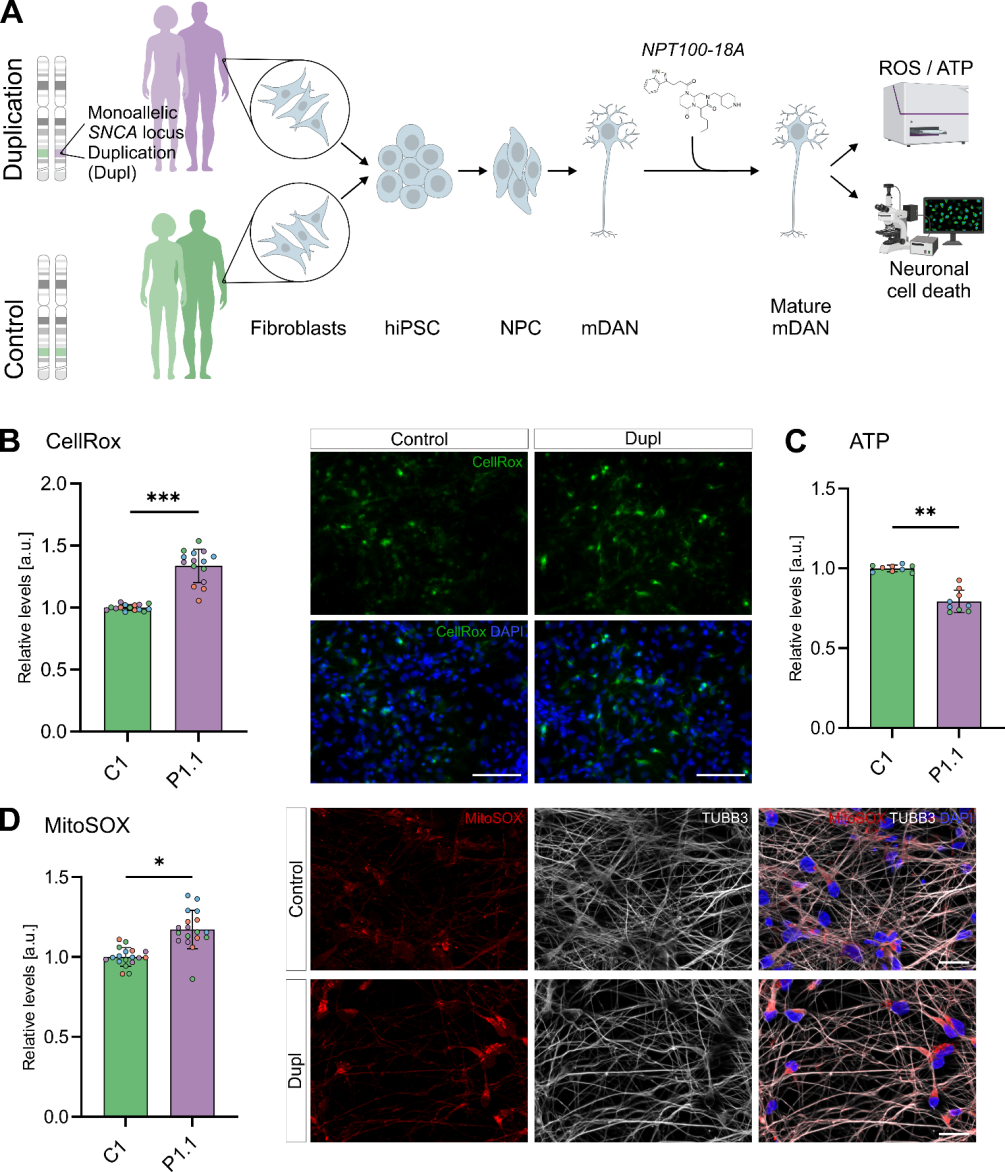
Increased oxidative stress and mitochondrial dysfunction in patient-derived mDANs. **(A)** Schematic of experimental design. Fibroblasts from PD patients carrying a monoallelic *SNCA* locus duplication and unaffected control individuals were obtained through punch biopsy of the skin. Fibroblasts were reprogrammed into human iPSCs. Human iPSCs were first differentiated into NPCs through dual SMAD inhibition and activation of canonical Wnt signaling and then into mDANs using a FGF8b-based protocol. mDANs were treated with NPT100-18A during the entire differentiation and maturation periods. **(B-D)** Relative levels depicted as fold changes from the mean of control cell line C1 ± SD of n wells from N independent differentiations. Replicates belonging to the same differentiation are shown in the same color. **(B)** CellRox Green fluorescence intensities (FIs) were assessed in living mDANs. CellRox and DAPI FIs were measured at 80 sites per well in 96-well plates using a CLARIOstar plate reader. CellRox FIs were normalized to respective DAPI FIs. Dots representing single well means (*n* = 15, *N* = 4). Right panels show representative microscopy images of CellRox and DAPI fluorescence signals. **(C)** Significantly reduced relative ATP levels measured in patient-derived compared to control mDANs. Relative ATP levels were assessed in mDAN lysates using a luciferase-based assay and a LUMIstar Omega plate reader. Values for single wells were normalized to the frequency of viable neurons (determined with a viability staining in mDANs cultured in parallel under the same conditions). Dots represent single wells (*n* = 9, *N* = 3). **(D)** MitoSOX Red FIs were measured in living mDANs analogously to **(B)**. Dots represent single well means (*n* = 18, *N* = 4). Right panels show representative microscopy images of MitoSOX and DAPI fluorescence signals; for better visualization of neurons, β3-tubulin (TUBB3) was additionally immunofluorescently labelled. **(B-D)** Student’s t-test, *P < 0.05, ** *P* < 0.01, ***P < 0.001. Scale bar 50 μm in **(B)** and 20 μm in **(D)**

We have previously reported the correlation of increased αSyn aggregation and oligomer levels and increased OS in mDANs from a PD patient (P2) [23, 31]. Before testing the effects of NPT100-18A (Fig. 1A), we first investigated the vulnerability of mDANs in additional PD patient- and control-derived mDAN lines (P1.1 and C1) and further characterized their mitochondrial phenotype. Detailed analyses of αSyn expression and aggregation in these patient-derived lines have been reported in our previous work [23, 31, 40, 41]. In the present study, we confirmed increased level of aggregated αSyn conformers in Triton-X100-insoluble fraction of patient-derived mDAN lysates by using the αSyn conformation-specific antibody MJFR-14-6-4-2 and immunoblotting (Fig. S1A). Next, we measured FIs of ROS probes in patient- and control-derived mDANs using CellRox and MitoSOX fluorescent probes to assess overall intracellular ROS and mitochondrial superoxide, respectively. As previously reported, FIs were measured using a multi-mode microplate reader to ensure fast and simultaneous detection across multiple neuronal lines and conditions [31]. A significant increase of MitoSOX FIs in P1.1 neurons upon treatment with 300 µM H_2_O_2_ for 2 h confirms the sensitivity of the MitoSOX probe to changes in ROS concentrations (Fig. S2A). Overall CellRox FIs were 33.7% higher in patient- compared to control-derived mDANs (*P* < 0.001; Fig. 1B) with a concomitant increase of 17.2% in mitochondrial superoxide probe FIs in PD patient-derived mDANs (*P* < 0.05; Fig. 1D). After determining increased levels of both, total intracellular and mitochondrial ROS probe intensities in patient-derived mDANs, we next asked whether this might have an impact on mitochondrial function. Oxidative phosphorylation (OXPHOS) is a very well-characterized core mitochondrial function, known to be prone to changes of the intra-mitochondrial milieu and interference by αSyn oligomers [20, 25]. Consequently, we measured relative ATP levels as a proxy for OXPHOS using a luciferase-based assay. Relative ATP-related luciferase signals were significantly lower in patient- compared to control-derived mDANs (79.3% of control, *P* < 0.01; Fig. 1C).

Taken together, these results demonstrate a concomitant increase of αSyn aggregation and OS burden in PD patient-derived mDANs and suggest mitochondrial dysfunction to be an additional contributor to neuropathology in this human in vitro PD model

### NPT100-18A reduces mitochondrial ROS probe intensities in patient-derived mDANs

**Fig. 2:**
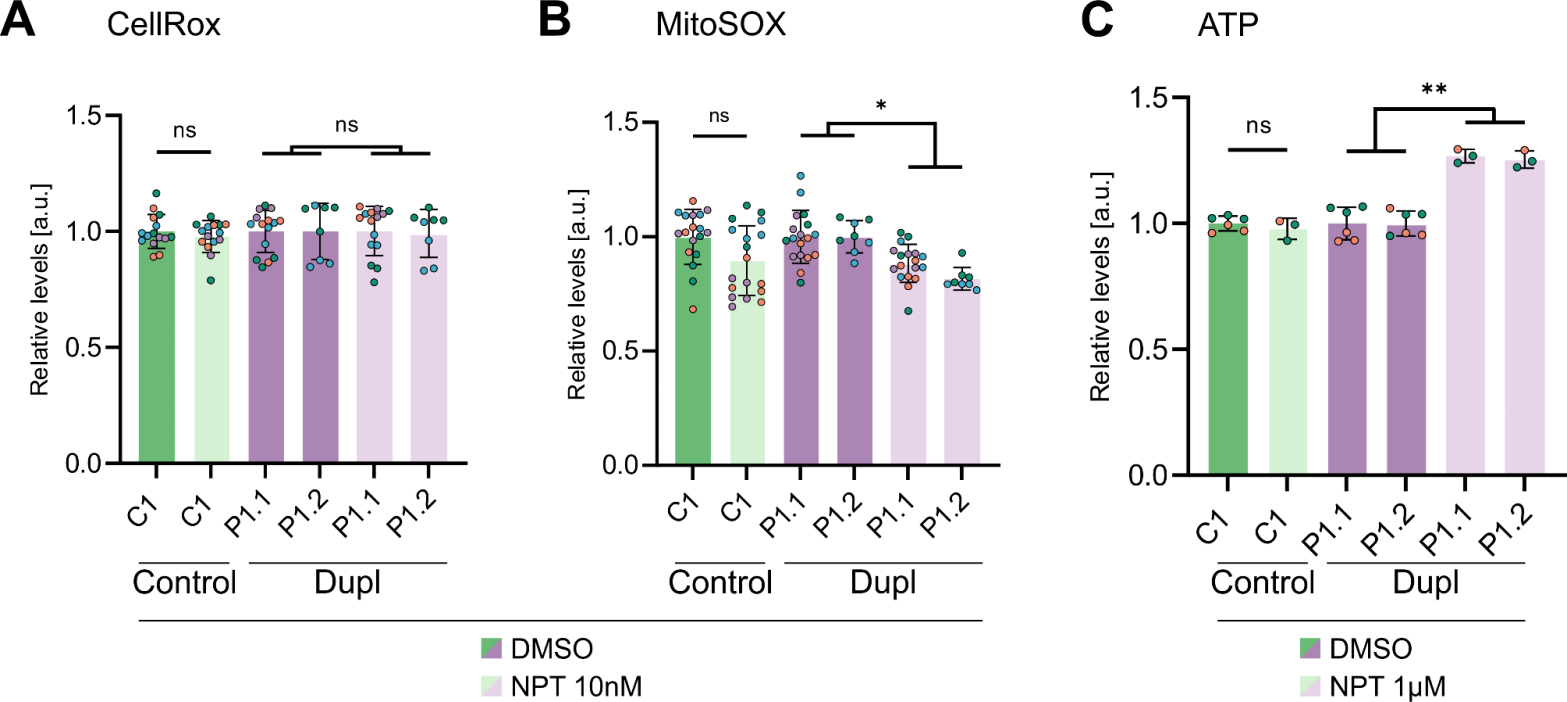
Treatment with NPT100-18A reduces MitoSOX fluorescence intensities (FIs) and, at higher dose, increases ATP luciferase signals. **(A-C)** Relative levels depicted as fold changes from the respective vehicle control ± SD of n wells from N independent differentiations. **(A)** Treatment with 10 nM NPT100-18A had no significant effect on CellRox FIs in patient- and control-derived mDANs. CellRox FI was quantified and depicted analogously to (Fig. 1B) with dots representing well means (*n* ≥ 8, *N* ≥ 2). **(B)** Treatment with 10 nM NPT100-18A significantly reduced MitoSOX FIs in patient-derived but not in control mDANs (*n* ≥ 8, *N* ≥ 2). **(C)** Treatment with 1 µM NPT100-18A significantly increased ATP-related luciferase signals in patient- and control-derived mDANs (*n* ≥ 3, *N* ≥ 2). **(A-C)** Student’s t-test for comparison of DMSO- and NPT100-18A-treated control neurons and nested t-test for comparison of DMSO- and NPT100-18A-treated patient lines, ns = not significant, *P < 0.05, **P < 0.01

After confirmation of elevated levels of aggregated αSyn conformers and demonstration of increased ROS probe levels and reduced relative ATP levels in the PD patient-derived mDAN line and, thus, characterizing a phenotype potentially amenable to treatment with NPT100-18A, we set out to investigate the effects of the novel small molecule on human iPSC-derived mDANs. Our treatment paradigm consisted of treatment with either 10 nM NPT100-18A or DMSO as a vehicle control during the entire differentiation and maturation period. NPT100-18A has previously been shown to reduce human αSyn aggregation in iPSC-derived neurons with *SNCA* duplication and other cellular PD models in vitro and in vivo [23, 32–34]. Here, we confirmed a trend towards reduced levels of Triton-X100-insoluble αSyn conformers upon treatment with NPT100-18A in both, patient- and control-derived mDANs (Fig. S1B). Measuring total intracellular ROS levels using CellRox fluorescent probe in living neurons, we did not find a significant effect of NPT100-18A- compared to DMSO-treatment, in either patient- or control-derived mDANs (Fig. 2A). Mitochondria are considered a major source of ROS production in neural cells and toxic oligomers of A53T mutant and WT αSyn have been shown to specifically increase mitochondrial oxidative damage [20, 42]. Therefore, we asked whether NPT100-18A, while not impacting intracellular CellRox FIs, may have a mitochondria-specific effect on ROS generation. To address this question, we measured mitochondrial superoxide in living mDANs as described above using the MitoSOX fluorescent probe. Treatment with 10 nM NPT100-18A indeed significantly reduced MitoSOX FIs compared to vehicle control (85.8% of control, *P* = 0.0493, nested t-test; Fig. 2B). Importantly, MitoSOX probe intensities of control-derived mDANs were not significantly affected by the NPT100-18A treatment. To assess whether NPT100-18A could mitigate mitochondrial dysfunction, we next measured relative ATP levels using the luciferase-based assay previously described. Treatment with 10 nM NPT100-18A did not significantly alter ATP-related luciferase levels (Fig. S2B). Since ATP is a downstream target influenced by various factors, we hypothesized that higher NPT100-18A concentrations might more effectively inhibit toxic αSyn aggregation and consequently impact mitochondrial function. We conducted a dose-response experiment with additional NPT100-18A concentrations (100 nM and 1 µM), finding a significant increase of ATP-related luciferase signals in patient-derived mDANs treated with 1 µM NPT100-18A (Fig. S2B). This effect was replicated in mDANs derived from a second patient iPSC clone P1.2 (Fig. 2C; pooled analysis for P1.1 and P1.2: +26.1% compared to DMSO, *P* = 0.0085, nested t-test), a response not observed in control-derived neurons.

### NPT100-18A rescues increased rates of neuronal cell death in patient-derived mDANs

**Fig. 3:**
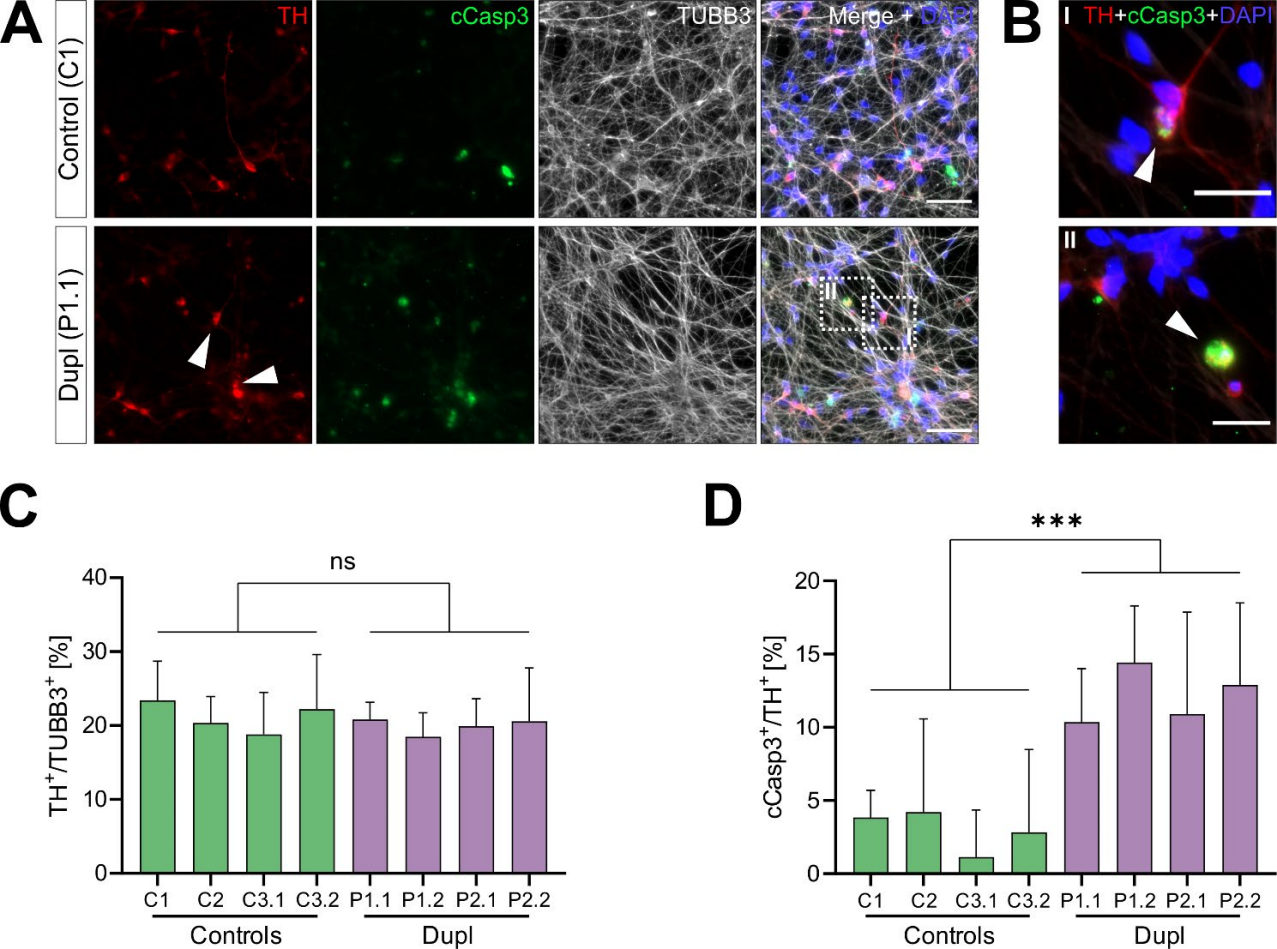
PD patient-derived mDANs show increased caspase-3 activation. **(A)** Untreated iPSC-derived mDANs (TUBB3^+^/TH^+^) from patients and controls were stained for cleaved caspase-3 (cCasp3) for evaluation of early neuronal cell death. Representative images used for quantifications shown in **(C-D)**. Arrows indicate TUBB3^+^/TH^+^/cCasp3^+^ neurons. **(B)** Enlarged views of selected cells marked by white frames in **(A)**, showing a neuron considered as in an early cell death stage TUBB3^+^/TH^+^/cCasp3^+^ (I) and a cCasp3^+^ structure not considered as a dying neuron (II). **(C-D)** Immunocytochemistry quantification. mDANs from two PD patients (with two different iPSC clones for each patient: P1.1, P1.2 and P2.1, P2.2) and three control individuals (with one iPSC clone for control 1 [C1] and 2 [C2] and two clones for C3: C3.1 and C3.2) were analyzed. **(C)** No significant difference in pooled rates of TH/TUBB3-double positive neurons was observed between patient and control cultures. Two-tailed nested t-test *P* = 0.46; mean control = 20.9%, mean patient = 19.9%. **(D)** Patient-derived mDANs exhibit significantly higher neuronal cCasp3 rates than controls. Nested t-test ***P = 0.0008; mean control = 3.68%, mean patient = 12.14%. Values are shown as mean ± SD of three independent differentiations. Scale bar 50 μm in **(A)** and 20 μm in **(B)**

**Fig. 4:**
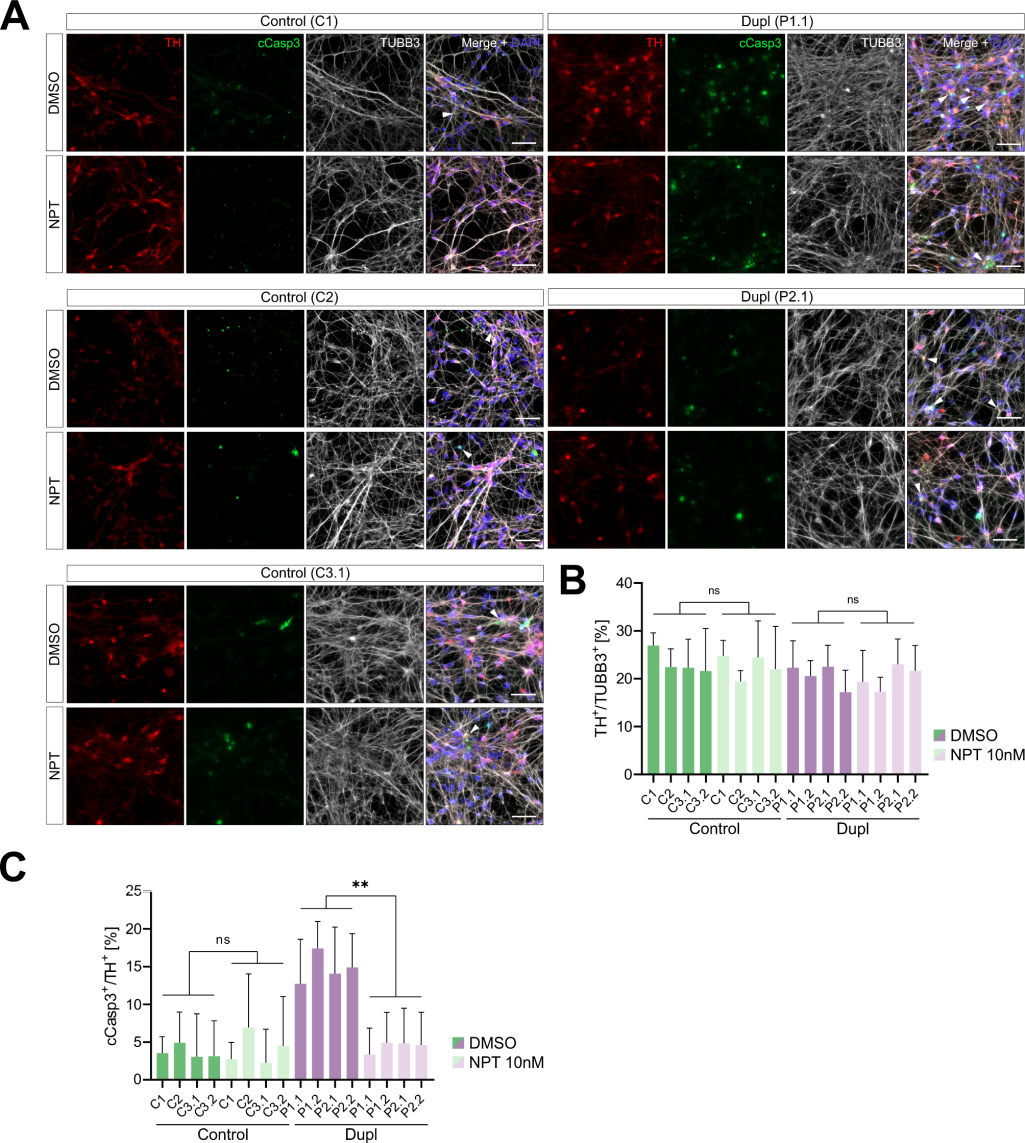
Increased caspase-3 activation in PD patient-derived mDANs is reversed by treatment with NPT100-18A. **(A)** NPT100-18A- and DMSO-treated iPSC-derived mDANs (TUBB3^+^/TH^+^) from PD patients and controls were stained for cleaved caspase-3 (cCasp3) to evaluate rates of early neuronal cell death. Representative images used for quantification shown in **(C)**. Scale bar 50 μm. **(B)** Immunocytochemistry quantification. mDANs from two PD patients (P) (with two different iPSC-clones for each patient) and three control individuals (with one iPSC clone for control [C] 1 and 2 and two clones for C3). No significant difference in pooled rates of TH/TUBB3-double positive neurons was observed between DMSO- and NPT100-18A-treated patient and control cultures, respectively. Control: two-tailed nested t-test *P* = 0.77; mean DMSO = 22.8%, mean NPT100-18A = 22.3%. Patient: two-tailed nested t-test *P* = 0.94; mean DMSO = 20.5%, mean NPT100-18A = 20.4%. **(C)** Treatment with 10 nM NPT100-18A significantly reduced rates of cCasp3-positive mDANs compared to vehicle condition in all patient-derived but not in control mDAN lines. Control: two-tailed nested t-test *P* = 0.86; mean DMSO = 3.7%, mean NPT100-18A = 3.9%. Patient: two-tailed nested t-test *P* = 0.004; mean DMSO = 14.4%, mean NPT100-18A = 4.2%; **P = 0.004. Values are shown as mean ± SD of three independent differentiations. Scale bar 50 μm

After determining the protective effects of NPT100-18A on intramitochondrial ROS, we next aimed to assess the compound’s capacity to protect patient-derived mDANs from αSyn-mediated neurodegeneration. To evaluate the efficacy of NPT100-18A treatment on preventing loss of mDANs, we first established a baseline of an early mediator of neuronal cell death in untreated patient- and control-derived neurons. For this, we performed immunocytochemistry for cCasp3, an established marker of early neuronal cell death [43], in neurons double-positive for β3-tubulin (TUBB3) and TH (Fig. 3A-B and Fig. S3A). To more comprehensively assess the ability of NPT100-18A to rescue neuronal degeneration, we included additional cell lines into the following analyses. IPSC-derived mDANs from two PD patients with *SNCA* locus duplication (two iPSC-clones per patient; Table 1) and three control individuals (one iPSC-clone for C1 and C2, and two iPSC-clones for C3; Table 1) were examined. Relative numbers of TH/TUBB3-double positive neurons were not significantly different between control and patient neurons (Fig. 3C; pooled percentages: control = 20.9%, patient = 18.7%, *P* = 0.16, nested t-test), confirming comparable differentiation efficiencies. The pooled mean percentage of dying mDANs (defined as cCasp3/TH-double positive) was, in contrast, significantly higher in patient- compared to control-derived mDANs (11.9% vs. 3.2%, respectively, nested t-test, *P* = 0.0003; Fig. 3D). Next, we evaluated the effects of NPT100-18A-treatment on neuronal caspase-3 activation. MDANs were treated with either 10 nM NPT100-18A or DMSO starting on the first day of differentiation and the rate of early neuronal cell death was assessed after 21 days of treatment. While no significant changes in the overall rates of TH/TUBB3-double positive neurons were observed (Fig. 4A-B), treatment with NPT100-18A significantly reduced the rates of cCasp3/TH-double positive dying neurons compared to DMSO-treatment in mDANs from both patients (pooled rates: 4.2% vs. 14.4%, nested t-test, *P* = 0.0041; Fig. 4A and C and Fig. S3B). NPT100-18A-treatment of patient-derived mDANs reduced the percentage of cCasp3/TH-double positive neurons to the level of DMSO-treated control-derived mDANs (pooled mean mDAN death rate 4.1% vs. 3.7%, respectively, nested t-test, *P* = 0.57; Fig. 4C) and had no significant effect in control-derived mDANs (Fig. 4C).

**Table 1:** Probands and iPSC lines

Proband	Abbreviation cell line	iPSC line name	Gender	Ethnicity	Age at biopsy (y)	Diagnosis	Genotype	Reference
**P1**	P1.1	CSC1-A	Female	Caucasian	53	Early-onset PD	Monoallelic *SNCA* locus duplication	[31, 35, 36]
	P1.2	CSC1-D						
**P2**	P2.1	SDi1-R-C3	Female	Caucasian	58	Early-onset PD	Monoallelic *SNCA* locus duplication	[23, 35, 36, 53]
	P2.2	SDi1-R-C11						
**C1**	C1	UKERi82A-S1_017	Female	Caucasian	66	Unaffected control	*SNCA* wildtype	[36]
**C2**	C2	UKERi33Q-R1-06	Female	Caucasian	45	Unaffected control	*SNCA* wildtype	[23, 31, 36, 53]
**C3**	C3.1	UKERiO3H-R1-001	Male	Caucasian	71	Unaffected control	*SNCA* wildtype	[23, 31, 36, 53, 54]
	C3.2	UKERiO3H-R1-005						

**Fig. 5:**
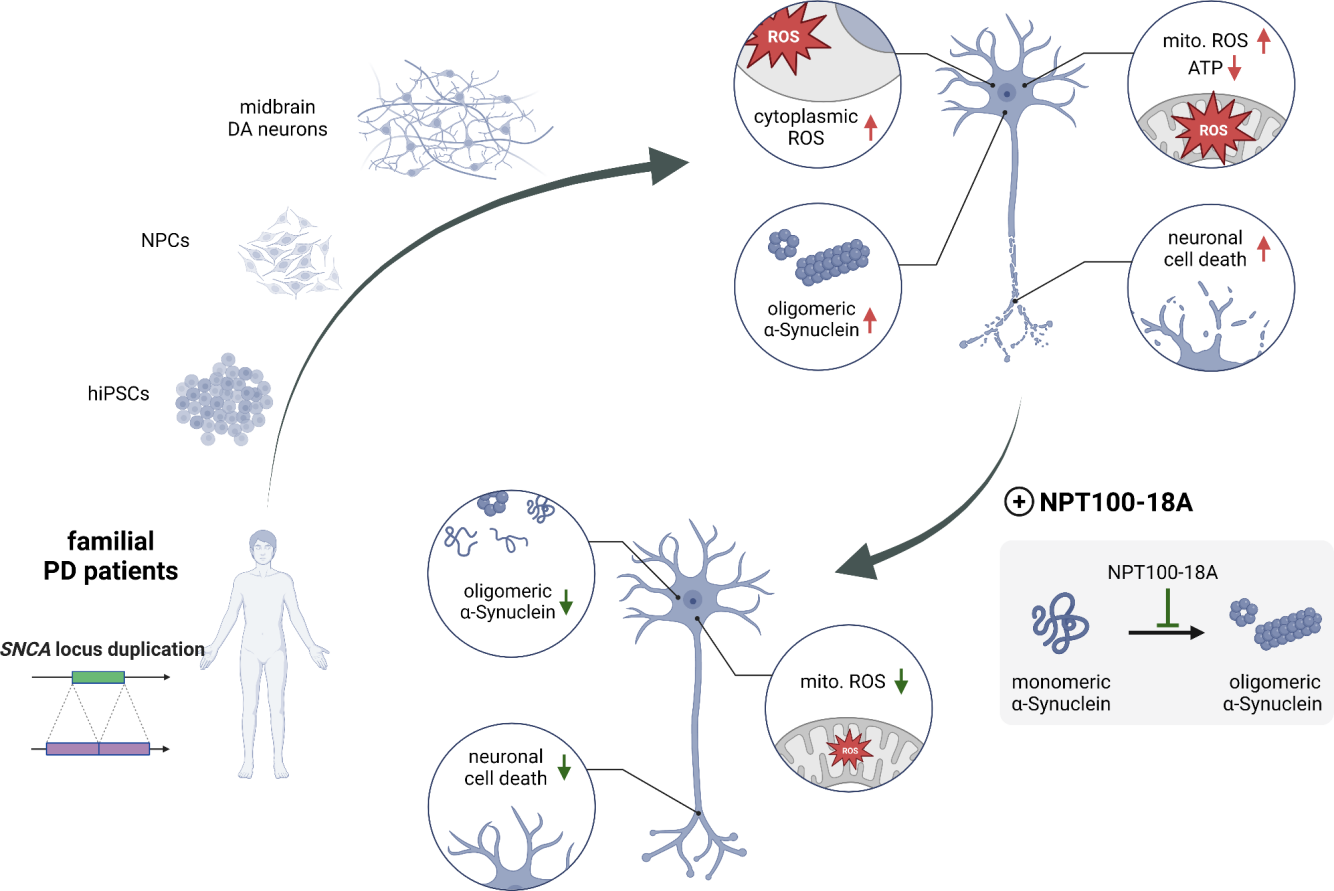
Graphical abstract of the study. Human midbrain dopaminergic (DA) neurons (mDANs) from patients with a monoallelic *SNCA* locus duplication were differentiated from induced pluripotent stem cells (iPSC) and neuronal precursor cells (NPCs). Increased aggregated αSyn conformer levels, including toxic oligomeric αSyn conformers (oligomeric α-Synuclein), increased signals of reactive oxygen species (ROS) probes in both cytoplasm and mitochondria (Mito.), mitochondrial dysfunction (measured by relative ATP levels), and increased caspase-3 activation in neurons (reflecting early neuronal cell death) were determined in human iPSC-derived mDANs from patients with a monoallelic *SNCA* locus duplication, recapitulating neuropathological hallmarks of PD. Treatment of mDANs with the αSyn misfolding inhibitor NPT100-18A, ameliorating αSyn aggregation, specifically reduces mitochondrial ROS probe levels and rescues caspase-3 activation in patient-derived neurons, thereby revealing the efficacy of the misfolding inhibitor in limiting loss of mDANs in PD

Altogether, these results reveal increased levels of caspase-3 activation, reflecting an early phase of an apoptotic cascade, in PD patient-derived mDANs and demonstrate that NPT100-18A limits the initiation of the neuronal cell death pathway caused by increased αSyn aggregation, essentially reducing the frequencies of dying mDANs to those of untreated control neurons (Fig. 5).

## Discussion

The peptidomimetic small molecule NPT100-18A and its derivatives pose a promising, new and causal therapeutic approach for PD. By inhibiting protein-protein interaction between the C-termini of αSyn monomers, the compound prevents the formation of toxic αSyn oligomers [32]. In this study, we set out to examine the effects of NPT100-18A on human iPSC-derived mDANs from PD patients with *SNCA* locus duplication. Confirming our previous findings of higher levels of aggregated αSyn conformers in *SNCA* locus duplication mDANs from an additional PD patient and establishing increased ROS probe levels, reduced ATP signals, and increased caspase-3 activation, we recapitulate neuropathological hallmarks of PD in human iPSC-derived mDANs from a patient with monoallelic *SNCA* locus duplication and characterize a phenotype potentially amenable to treatment with the αSyn misfolding inhibitor. In a set of exploratory experiments, we subsequently demonstrate that treatment with NPT100-18A reduces mitochondrial ROS probe intensities, increases ATP-related signals, and rescues neuronal cell death initiation in patient-derived lines. We thereby show for the first time the efficacy of the αSyn misfolding inhibitor in reducing the number of apoptotic mDANs in a human iPSC-derived model of PD and provide a first mechanistic insights into how a mitochondria-specific antioxidant effect might elicit its neuroprotective effects.

Recent studies have revealed a causal link between neurotoxic αSyn oligomers, disruption of normal mitochondrial function, and oxidative stress at several levels. ASyn oligomers have been shown to interfere with complex I of the electron transport chain (ETC) and ATP synthase and thereby directly induce mitochondrial bioenergetic defects [20, 25]. In line with this, in the present study, we found significantly reduced ATP-related signals in patient-derived mDANs with monoallelic *SNCA* locus duplication with the higher presence of aggregated αSyn conformers. While mitochondrial dysfunction and reduced ATP regeneration pose a significant challenge to all neuronal subpopulations, mDANs seem to be particularly vulnerable due to the intertwining of monoamine oxidase (MAO)-dependent dopamine (DA) metabolism and ETC activity. Electrons released during degradation of DA, which under physiological conditions are shuttled into the mitochondrial intermembrane space and contribute to ATP regeneration, become available to form intramitochondrial and cytoplasmic ROS upon uncoupling of MAO and the ETC [44]. We previously reported increased ROS levels to be an important determinant of the cell type-specific vulnerability of mDANs in PD, differentiating them from other non-dopaminergic neuronal subpopulations, such as cortical projection neurons [31]. Here, we found both overall intracellular and mitochondrial ROS probe intensities to be significantly increased in patient-derived compared to control mDANs. Furthermore, αSyn oligomer-induced exacerbation of intramitochondrial ROS burden can result in opening of permeability transition pore (PTP) [20]. Opening of PTP triggers changes in mitochondrial membrane potential, calcium homeostasis, and release of cytochrome C, eventually leading to activation of executioner caspases and initiation of neuronal cell death [20, 45]. In accordance with this, in patient-derived mDANs, we observed significantly increased rates of neurons positive for cCasp3, an important executioner caspase and well-established marker for early neurodegenerative processes [46, 47]. While significantly higher rates of cCasp3-positive dopaminergic neurons contrast with the absence of differences in the frequency of TH/TUBB3-double positive neurons in patient-derived cultures, this discrepancy might be explained by cCasp3 being a very early mediator of neuronal apoptosis, whose activation does not invariably result in immediate neuronal cell death [47, 48]. Thus, an early caspase-3 activation might not yet result in detectable changes in the overall population of TH/TUBB3-double positive neurons within the relatively short experimental timeframe in the present study. In summary, we demonstrate that this model recapitulates pathological features deemed pivotal in the mDAN-specific neurodegenerative cascade in PD.

Considering that αSyn aggregation has been recognized as a key driver of neuropathology in PD and its intertwining with DA metabolism and mitochondrial dysfunction, we hypothesized that interference with αSyn aggregation might ameliorate the cellular phenotype characterized above and thereby prevent loss of patient-derived mDANs. Among the most promising inhibitors of αSyn aggregation is NPT100-18A, a small molecule which prevents αSyn oligomerization by blocking protein-protein interaction of αSyn monomers. NPT100-18A has been previously tested in αSyn transgenic rodent models of PD (primary cortical neurons with lentiviral αSyn overexpression and mThy1-WT-αSyn transgenic mice) and an A53T-mutant αSyn-expressing iPSC-derived model of PD [32, 34]. In these models, treatment with the misfolding inhibitor reduced αSyn aggregation and ameliorated morphological defects of neurites in vitro as well as behavioral deficits in vivo [32, 34]. In addition, we previously demonstrated reduced αSyn oligomerization and rescue of mitochondrial axonal transport defects in iPSC-derived mDANs transduced with mutant or WT αSyn upon NPT100-18A treatment [23]. However, to date, no comprehensive characterization of the downstream mechanisms of action of NPT100-18A on a cellular level in human neurons with physiologically increased WT αSyn expression has been reported.

To fill the gap in knowledge of NPT100-18A effects in human dopaminergic neurons, we chose iPSC-derived mDANs from patients with monoallelic *SNCA* locus duplication as a WT αSyn overexpressing model of PD. Since the inhibitory effects of NPT100-18A on αSyn aggregation have been thoroughly characterized in various models and contexts by us and others, and are established at the molecular level [23, 32], we concentrated here on further exploration of potential downstream effects of αSyn aggregation reduction mediated by this small molecule in neurons. Using fluorescent probes in living mDAN cultures, revealed a significant reduction of mitochondrial ROS probe levels in treated patient-derived neurons, while no effect on overall intracellular ROS probe levels was noticeable. In line, we observed significantly increased ATP-related luminescence levels upon treatment with a higher dose of NPT100-18A. These analyses are, however, exploratory in nature and warrant further validation in future studies using both additional patient-derived neuronal lines as well as a larger array of markers of mitochondrial function. To further test whether the observed reduction in αSyn aggregation could prevent initiation of apoptosis in human dopaminergic neurons in vitro, we performed immunocytochemical staining, which revealed a significantly reduced rate of cCasp3-positive neurons in NPT100-18A-treated patient-derived mDANs. Notably, treated patient-derived mDANs showed rates of cCasp3-positive neurons comparable to those of control-derived mDANs.

While our study demonstrates that NPT100-18A treatment reduces mitochondrial ROS probe intensities, increases ATP-related signals, and prevents initiation of neuronal cell death in patient-derived mDANs, the timing of compound administration warrants further reflection. αSyn aggregation initiates acute cellular stress responses, including OS and mitochondrial dysfunction, which progressively contribute to neuronal degeneration. Administering the compound from the first day of neuronal differentiation in vitro allowed us to evaluate its effects on early stress mechanisms and their downstream consequences. This treatment strategy was already successfully applied in our previous study elucidating an influence of αSyn aggregates on mitochondrial axonal transport in human iPSC-derived *SNCA* locus duplication neurons [23]. However, this experimental approach does not directly model the therapeutic application in patients, where intervention would occur at later stages of disease progression in mature neurons. Our findings thus uncover new downstream effects of the compound and provide proof-of-concept evidence for the compound’s neuroprotective potential, but require further validation in experimental paradigms mimicking late-stage pathology in mDAN cultures maintained for much longer periods. At the same time, our findings may hold potential for advancing other novel therapeutic strategies, such as transplanting iPSC-derived dopaminergic progenitors into the brains of PD patients to replace degenerated neurons [49]. Combining this approach with e.g. NPT200-11 treatment could provide additional support to developing transplanted neurons, potentially enhancing their survival and integration.

 Degeneration of mDANs and subsequent disruption of nigrostriatal circuits is considered one of the most important pathological hallmarks of PD and causative for the development of disabling motor symptoms [50–52]. Here, we demonstrate that NPT100-18A treatment can limit the number of early apoptotic dopaminergic neurons in a human in vitro model of PD.

## Conclusions

In conclusion, we demonstrate that administration of αSyn misfolding inhibitor NPT100-18A limits an initiation of neuronal cell death of mDANs in a human in vitro model of PD. In addition, we provide a first mechanistic insight into how a compartment-specific antioxidant effect in mitochondria might mediate the neuroprotective effects of NPT100-18A in human dopaminergic neurons.

## Electronic supplementary material

Below is the link to the electronic supplementary material.

**Figure S1.** Analyses of αSyn conformers and treatment with NPT100-18A in iPSC-derived mDANs. **(A)** Dot blot panels and quantitative analysis of αSyn shows increased total αSyn and αSyn conformers in Triton-X100-insoluble fraction of midbrain dopaminergic neuron (mDAN) lysates from PD patient with monoallelic *SNCA* locus duplication (Dupl). **(B)** Dot blot panels and quantitative analysis of αSyn shows lower levels of Triton-X100-insoluble αSyn conformers upon treatment with NPT100-18A (NPT).

**Figure S2.** MitoSOX assay sensitivity and NPT100-18A dose response for ATP levels in mDANs. **(A)** Relative MitoSOX probe levels depicted as fold changes of the untreated neurons without H_2_O_2_ challenge ± SD. In both DMSO-treated and untreated neurons, derived from the PD patient line P1.1, MitoSOX fluorescence intensities (FIs) are significantly increased after a 2-hour (2h) 300µM H_2_O_2_ challenge, confirming the assay’s sensitivity to changes in ROS levels. **(B)** Dose response for relative ATP luciferase levels in control (line C1) and patient-derived mDANs (line P1.1) after treatment with 10 nM, 100 nM, and 1 μM of NPT100-18A (NPT). Two-way ANOVA with Tukey’s post-hoc test for multiple comparisons; ns = not significant, *P < 0.05, **P < 0.01, ****P < 0.0001.

**Figure S3.** Neuronal caspase-3 activation and treatment with NPT100-18A in iPSC-derived mDANs. **(A)** IPSC-derived mDANs (TUBB3^+^/TH^+^) from patients (Dupl) and controls were stained for cleaved Caspase-3 (cCasp3) for evaluation of early neuronal cell death. Representative images used for the quantification in Fig. 3C-D. mDANs from two PD patients (one iPSC clone for patient 1 [P1.2] and two clones for patient 2: P2.1, P2.2) and two control individuals (with one iPSC clone for control 2 [C2] and two clones for control 3: C3.1 and C3.2) are shown. **(B)** NPT100-18A- and DMSO-treated iPSC-derived mDANs (TUBB3+/TH+) from PD patients (Dupl) and controls were stained for cCasp3 to evaluate neuronal cell death rates. Representative images from two PD patients (P1.2 and P2.2) and two control individuals (C2 and C3.2) are shown. Scale bar 50μm.

Supplementary Material 4

## Data Availability

Data is provided within the manuscript or supplementary information files. All data are also available from the corresponding author upon reasonable request.

## Abbreviations

ATP: Adenosine triphosphate
BDNF: Brain-derived neurotrophic factor
C: Control
cCasp3: Cleaved caspase-3
DA: Dopamine
DAPI − 4′,6: Diamidino-2-phenylindole
DPBS: Dulbecco’s phosphate-buffered saline
ER: Endoplasmic reticulum
ETC: Electron transport chain
FGF8: Fibroblast growth factor 8
GDNF: Glial cell-line derived neurotrophic factor
h: Hours
HRP: Horseradish peroxidase
iPSC: Induced pluripotent stem cell
MAO: Monoamine oxidase
mDANs: Midbrain dopaminergic neurons
min: Minutes
NPCs: Neural precursor cells
OS: Oxidative stress
P: Patient
PD: Parkinson’s disease
PFA: Paraformaldehyde
PMA: Purmorphamine
PTP: Permeability transition pore
ROS: Reactive oxygen species
RT: Room temperature
*SNCA*: αSyn gene
TBS: Tris-buffered saline
TBST: TBS containing 0.1% Tween 20
TGF: β3-transforming growth factor beta-3
TH: Tyrosine hydroxylase
TUBB3: β3-tubulin
WT: Wild-type
αSyn: Alpha-synuclein
