# Supplementary figures and images for: NPT100-18A rescues mitochondrial oxidative stress and neuronal degeneration in human iPSC-based Parkinson’s model
Source: BMC Neurosci. 2025 Jan 28;26:8. doi: 10.1186/s12868-025-00926-y (PMC11773751; doi:10.1186/s12868-025-00926-y)

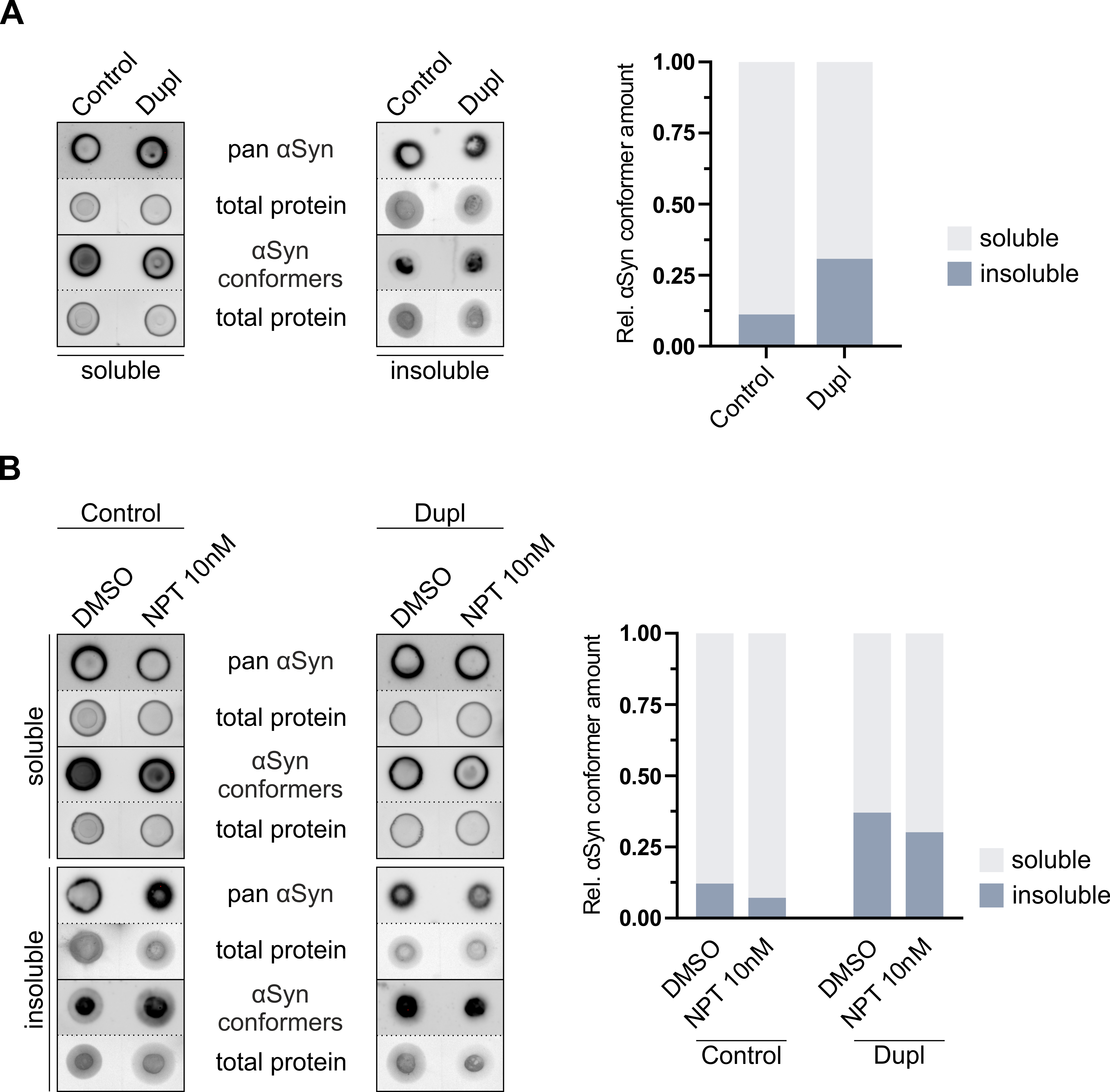

Supplement: Supplementary file 1 — Figure S1. Analyses of αSyn conformers and treatment with NPT100-18A in iPSC-derived mDANs. (A) Dot blot panels and quantitative analysis of αSyn shows increased total αSyn and αSyn conformers in Triton-X100-insoluble fraction of midbrain dopaminergic neuron (mDAN) lysates from PD patient with monoallelic SNCA locus duplication (Dupl). (B) Dot blot panels and quantitative analysis of αSyn shows lower levels of Triton-X100-insoluble αSyn conformers upon treatment with NPT100-18A (NPT). [file 12868_2025_926_MOESM1_ESM.tif]

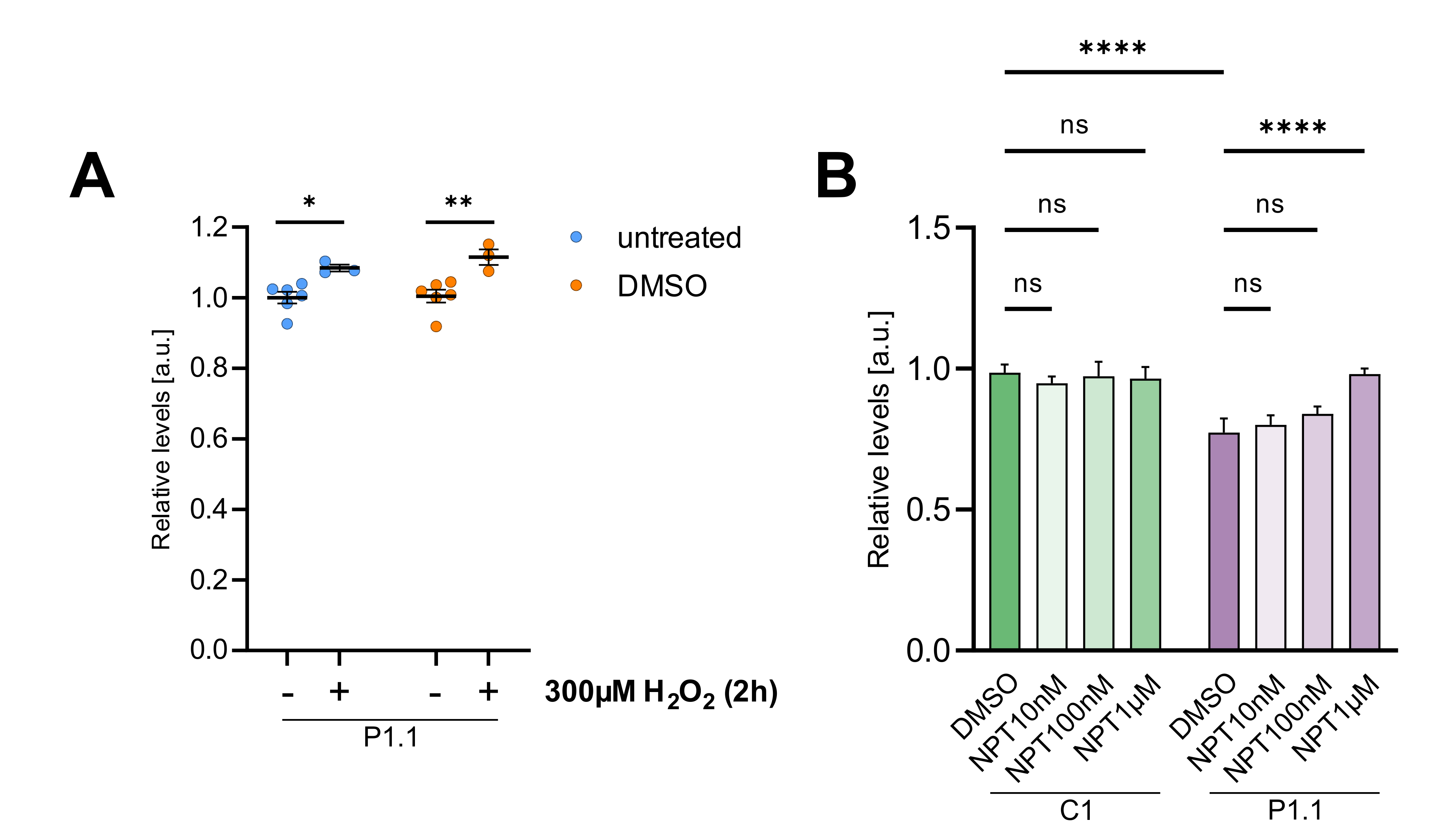

Supplement: Supplementary file 2 — Figure S2. MitoSOX assay sensitivity and NPT100-18A dose response for ATP levels in mDANs. (A) Relative MitoSOX probe levels depicted as fold changes of the untreated neurons without H2O2 challenge ± SD. In both DMSO-treated and untreated neurons, derived from the PD patient line P1.1, MitoSOX fluorescence intensities (FIs) are significantly increased after a 2-hour (2h) 300µM H2O2 challenge, confirming the assay’s sensitivity to changes in ROS levels. (B) Dose response for relative ATP luciferase levels in control (line C1) and patient-derived mDANs (line P1.1) after treatment with 10 nM, 100 nM, and 1 μM of NPT100-18A (NPT). Two-way ANOVA with Tukey’s post-hoc test for multiple comparisons; ns = not significant, *P < 0.05, **P < 0.01, ****P < 0.0001. [file 12868_2025_926_MOESM2_ESM.tif]

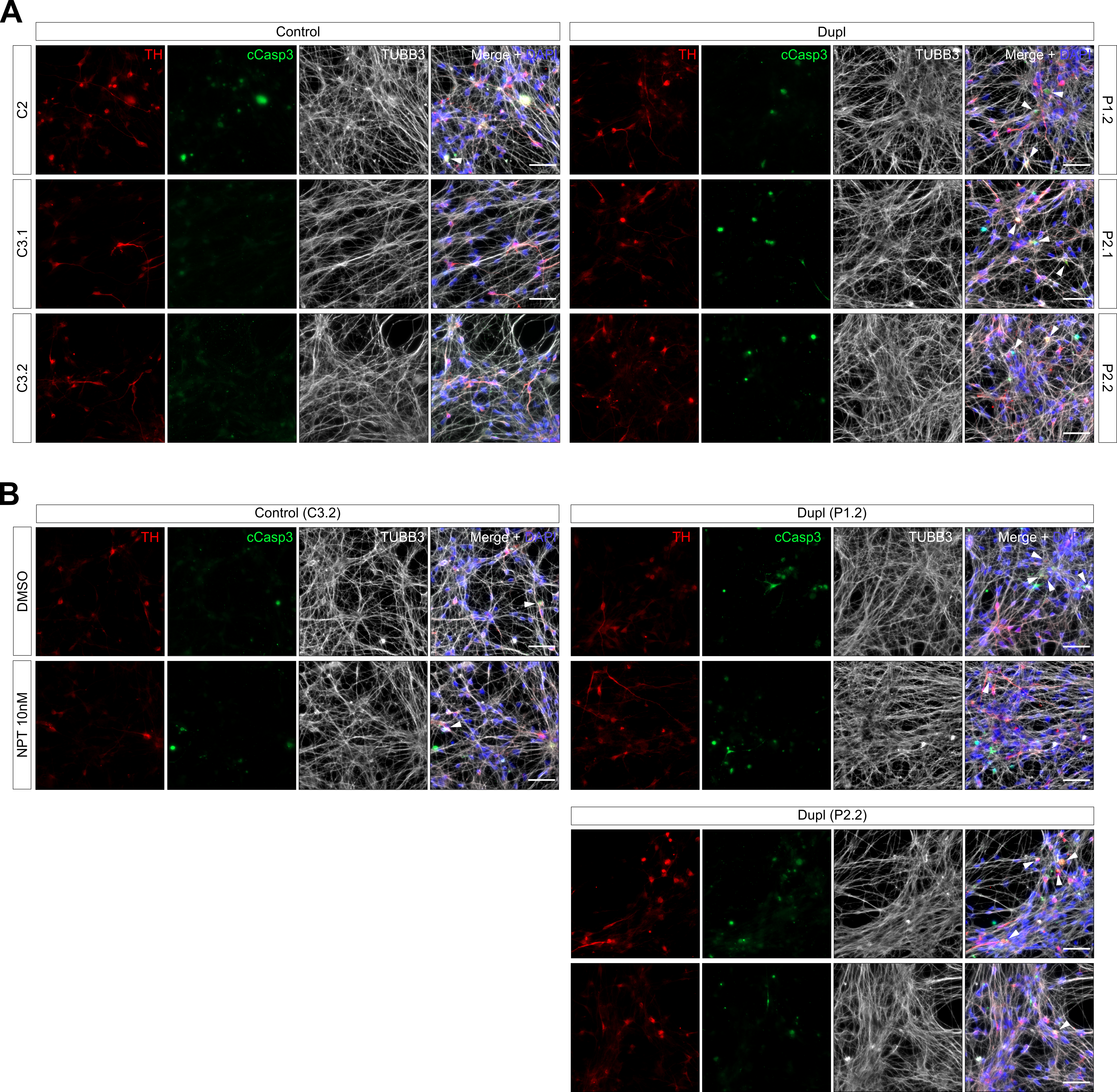

Supplement: Supplementary file 3 — Figure S3. Neuronal caspase-3 activation and treatment with NPT100-18A in iPSC-derived mDANs. (A) IPSC-derived mDANs (TUBB3+/TH+) from patients (Dupl) and controls were stained for cleaved Caspase-3 (cCasp3) for evaluation of early neuronal cell death. Representative images used for the quantification in Fig. 3C-D. mDANs from two PD patients (one iPSC clone for patient 1 [P1.2] and two clones for patient 2: P2.1, P2.2) and two control individuals (with one iPSC clone for control 2 [C2] and two clones for control 3: C3.1 and C3.2) are shown. (B) NPT100-18A- and DMSO-treated iPSC-derived mDANs (TUBB3+/TH+) from PD patients (Dupl) and controls were stained for cCasp3 to evaluate neuronal cell death rates. Representative images from two PD patients (P1.2 and P2.2) and two control individuals (C2 and C3.2) are shown. Scale bar 50μm. [file 12868_2025_926_MOESM3_ESM.tif]
