# Supplementary material for: NPT100-18A rescues mitochondrial oxidative stress and neuronal degeneration in human iPSC-based Parkinson’s model
Source: BMC Neurosci. 2025 Jan 28;26:8. doi: 10.1186/s12868-025-00926-y (PMC11773751; doi:10.1186/s12868-025-00926-y)
Supplement: Supplementary file 4 — Supplementary Material 4 [file 12868_2025_926_MOESM4_ESM.docx]

|  | **Reagent** | **Manufacturer** | **Cat#** | **RRID** | **Dilution/**  **Concentration** |
| --- | --- | --- | --- | --- | --- |
| *Antibodies & dyes* | α-Synuclein [Syn1] | BD Biosciences | 610787 | AB_398108 | 1:500 |
|  | α-Synuclein aggregate [MJFR-14-6-4-2] | Abcam | ab209538 | AB_2714215 | 1:500 |
|  | β-Actin | Sigma-Aldrich | A5441 | AB_476744 | 1:5000 |
|  | β3-Tubulin (DB^#^) | Covance | MMS-435P | AB_2313773 | 1:1000 |
|  | β3-Tubulin (ICC^#^) | BioLegend | 801201 | AB_2313773 | 1:500 |
|  | Tyrosine hydroxylase (TH) | Santa Cruz Biotechnology | sc-7847 | AB_671396 | 1:500 |
|  | cleaved Caspase 3 (cCasp3) | Cell Signaling Technology | 9664 | AB_2070042 | 1:500 |
|  | HRP anti-rabbit IgG | Thermo Fisher Scientific | 32260 | AB_1965959 | 1:10000 |
|  | HRP anti-mouse IgG | Thermo Fisher Scientific | G-21040 | AB_2536527 | 1:10000 |
|  | AF488 anti-rabbit IgG | Thermo Fisher Scientific | A21206 | AB_2535792 | 1:500 |
|  | AF568 anti-goat IgG | Thermo Fisher Scientific | A11057 | AB_2534104 | 1:500 |
|  | AF647 anti-mouse | Thermo Fisher Scientific | A32787 | AB_2762830 | 1:500 |
|  | CellRox Green | Thermo Fisher Scientific | C10444 | N/A | 5 µM |
|  | MitoSOX Red | Thermo Fisher Scientific | M36008 | N/A | 5 µM |
|  | CellTiter-Glo 2.0 | Promega | G9241 | N/A | N/A |
|  | Image-IT DEAD Green | Thermo Fisher Scientific | I10291 | N/A | 100 nM |
|  | DAPI | Sigma-Aldrich | D9542 | N/A | 1 or 10 µg/ml |
| *ICC & WB* | Paraformaldehyde (PFA) | Sigma-Aldrich | P6148 | N/A | 4% |
|  | Normal donkey serum | Sigma-Aldrich | D9663 | AB_2810235 | 5% |
|  | Bovine serum albumin | Sigma-Aldrich | A7906 | N/A | 3% |
|  | Aqua Polymount | Polysciences | 18606 | N/A | N/A |
|  | cOmplete Protease Inhibitor Cocktail | Roche | 04693116001 | N/A | N/A |
|  | Pierce BCA Protein Assay Kit | Thermo Fisher Scientific | 23225 | N/A | N/A |
|  | Urea | Sigma-Aldrich | U5378 | N/A | 500 mM |
|  | Sodium dodecyl sulfate (SDS) | Sigma-Aldrich | L3771 | N/A | 5% |
|  | Nitrocellulose membrane | Bio-Rad Laboratories | 1620112 | N/A | N/A |
|  | SuperSignal West | Thermo Fisher Scientific | 34577 | N/A | N/A |
|  | Dulbecco′s Phosphate Buffered Saline (DPBS) | Thermo Fisher Scientific | 14190144 | N/A | N/A |
|  | DPBS +Ca^2+^/Mg^2+^ | Thermo Fisher Scientific | 14040133 | N/A | N/A |
| *Cell culture* | DMEM/F12 Medium | Thermo Fisher Scientific | 31331028 | N/A | N/A |
|  | Neurobasal Medium | Thermo Fisher Scientific | 21103049 | N/A | N/A |
|  | N2 Supplement | Thermo Fisher Scientific | 17502048 | N/A | N/A |
|  | B27 Supplement | Thermo Fisher Scientific | 12587010 | N/A | N/A |
|  | FGF-8b | Peprotech | 100–25 | N/A | 100 ng/ml |
|  | Purmorphamine (PMA) | Tocris | 4551 | N/A | N/A |
|  | Ascorbic acid | Sigma-Aldrich | PHR1008 | N/A | 200 nM |
|  | TGF-β3 | Peprotech | AF-100-36E | N/A | 1 ng/ml |
|  | GDNF | Peprotech | 450–10 | N/A | 10 ng/ml |
|  | BDNF | Peprotech | 450-02 | N/A | 10 ng/ml |
|  | dibutyryl-cAMP | AppliChem | A0455 | N/A | 500 µM |
|  | Geltrex | Thermo Fisher Scientific | A1413201 | N/A | N/A |
|  | DMSO | Sigma-Aldrich | D2438 | N/A | N/A |

^#^ DB – dot blot; ICC - immunocytochemistry
